# Soil erodibility indices under different land uses in Ri-Bhoi district of Meghalaya (India)

**DOI:** 10.1038/s41598-020-72070-y

**Published:** 2020-09-11

**Authors:** Manish Olaniya, Pradip Kumar Bora, Susanta Das, Pukhrambam Helena Chanu

**Affiliations:** 1 School of Natural Resource Management, College of Post-Graduate Studies in Agricultural Sciences, Central Agricultural University(Imphal), Umiam, Meghalaya 793103 India; 2 College of Agricultural Engineering and Technology, Punjab Agricultural University, Ludiana, Punjab 141027 India; 3 Institute of Agricultural Sciences, Banaras Hindu University, Varanasi, 221005 India

**Keywords:** Environmental sciences, Natural hazards, Solid Earth sciences, Engineering, Physics

## Abstract

In absence of soil erosion plots for determination of erodibility index (K) for erosion models like Universal Soil Loss Equation (USLE) or Revised Universal Soil Loss Equation (RUSLE) to estimate soil erosion, empirical relations are used. In the present study, soil erodibility index was determined for entire Ri-bhoi district of Meghalaya based on soil physical and chemical properties through empirical relationship and presented in a map form. Dominant land uses of the district were identified through geo-spatial tools which were viz. agriculture, forest, *jhum* land and wasteland. Soil samples from surface depth (01–15 cm) were collected from areas of different dominant land uses. Twenty five sampling points were selected under each land use type and geo-coded them on the base map of Ri-bhoi district. Apart from K-index, Clay Ratio, Modified Clay Ratio and Critical Soil Organic Matter were also determined for understanding the effect of primary soil particles on erodibility. In agriculture land use system K-index values were found in the range of 0.08–0.41 with an average of 0.25 ± 0.02. In case of *jhum*, forest and wasteland these were in the range of 0.08–0.42 with an average of 0.20 ± 0.01; 0.09–0.40 with an average of 0.22 ± 0.02, and 0.10–0.34 with an average value of 0.23 ± 0.02, respectively. Clay ratio (2.74) and Modified clay ratio (2.41) were observed to be higher in forest LUS, lower clay ratio (1.97) and modified clay ratio (1.81) were found in the wasteland indicating erosion susceptibility in forested area. The values of Critical Level of Organic Matter (CLOM) for the district ranged from 4.72 to 16.56. Out of 100 samples, only one sample had CLOM value less than 5 and rest 99 samples had values more than 5 indicating that the soils of the district had moderate to stable soil structure and offer resistance to erosion. All the indices values of geo-coded points were then interpolated in the Arc-GIS environment to produce land use based maps for Ri-bhoi district of Meghalaya. As K-index is a quantitative parameter which is used in models, the index can be then interpolated for estimation of soil erosion through USLE or RUSLE for any given situation.

## Introduction

Land, water and vegetation are the most precious resources especially for mountainous region, where the economy is based on subsistence agriculture and forestry. North Eastern Region (NER) of India is one such area, where the livelihood of people is largely dependent on agriculture. In Recent times, the demand of land has increased due to population growth, which in turn has led to massive clearing of vegetation cover for agricultural land that is found to be a major cause of soil degradation^1–3^. In India, land degradation is primarily caused due to inappropriate agricultural practices which brings upon adverse effect on livelihood^4^. Water erosion is the chief contributor of soil degradation worldwide. Soil erosion has negative impact specially on agricultural production, water resource quality and the ecosystem sustainability^5^. Among the major causes of soil degradation in India, water erosion is considered to be the most severe one which covers almost 68.39% of the affected area resulting into the annual soil loss of about 5.3 billion tons through erosion^6^. Further, it is also found that most of the hotspot of soil erosion have erosion rate more than 20 Mg/ha/y^1^. The traditional agricultural practices without conservation measures and excessive exploitation of resources have also caused severe soil erosion, landslides, siltation of rivers and flash flood in different part of North Eastern Region.

Estimation of soil erosion has always been a primary concern for ecologists and soil conservation workers. Most of workers use either USLE^7^ or RUSLE^8^. The equation is expressed as:

1 $${\text{A}} = {\text{R}}.{\text{K}}.{\text{L}}.{\text{S}}.{\text{C}}.{\text{P}}$$

wherein, A is the annual soil loss (Mg ha^−1^ year^−1^), R is rainfall erosivity (MJ mm ha^−1^ h^−1^ year^−1^), K is soil erodbility factor (Mg h MJ^−1^ mm^−1^), L is the slope length factor, S is the slope steepness factor, C is the cover management factor and P is conservation practice factor. Among these factors soil erodibility is considered to be a very complex factor and integrated effect of rainfall, runoff, and infiltration on soil^9^. In fact K is a factor having wide range of integrated parameters some of then only related to soil^10^. The erodibility factor is best determined from long time measurement of soil erosion from 22.1 m long and 9% steep erosion plots^11^. In absence of soil erosion plots K can be estimated through empirical relationships as developed by many researchers ^8, 12–16^. In the present study, we have planned to use empirical model as given by Renard et al.^8^.

In the empirical equation^8^ the physico-chemical properties of soil, viz*.,* primary particles, organic matter content, soil permeability and soil structure^8, 17, 18^ are considered. It is related to soil texture, organic matter, soil structure, pH and bulk density of plow layer and subsoil, steepness and concavity or convexity of slope, pore space filled by air, residual effects of sod crops, aggregation, parent material, and various interactions of these variables^19^. Soil aggregation and percentage of water stable aggregates are other information indicating soil erodibility. It has been reported that land use system has significant influence on soil aggregation^20^ which in turn affect on soil erodibility.

Apart from quantitative indices for soil erodibility to estimate soil erosion some other indices are also used by researchers to understand the proneness of soil to erosion. Among them, Dispersion Ratio (DR), Clay Ratio (CR)^21, 22^, Modified Clay Ratio (MCR)^6^ and Critical Level of Soil Organic Matter (CLOM)^23^ are found to be commonly used. Prioritization of soil conservation measures can also possible to do with the help of indices such as dispersion ratio, clay ratio, modified clay ratio and CLOM as reported for East Sikkim district of India^24^. In the present study, Clay Ratio, Modified Clay Ratio and Critical Soil Organic Matter and K-index were determined for the district of Ri-Bhoi in Meghalaya State of India. These indices are then presented in the map form by overlaying on the district map so that any worker can pick up the values for estimating soil erosion in a given land use.

## Materials and methods

### Study area

**Figure 1:**
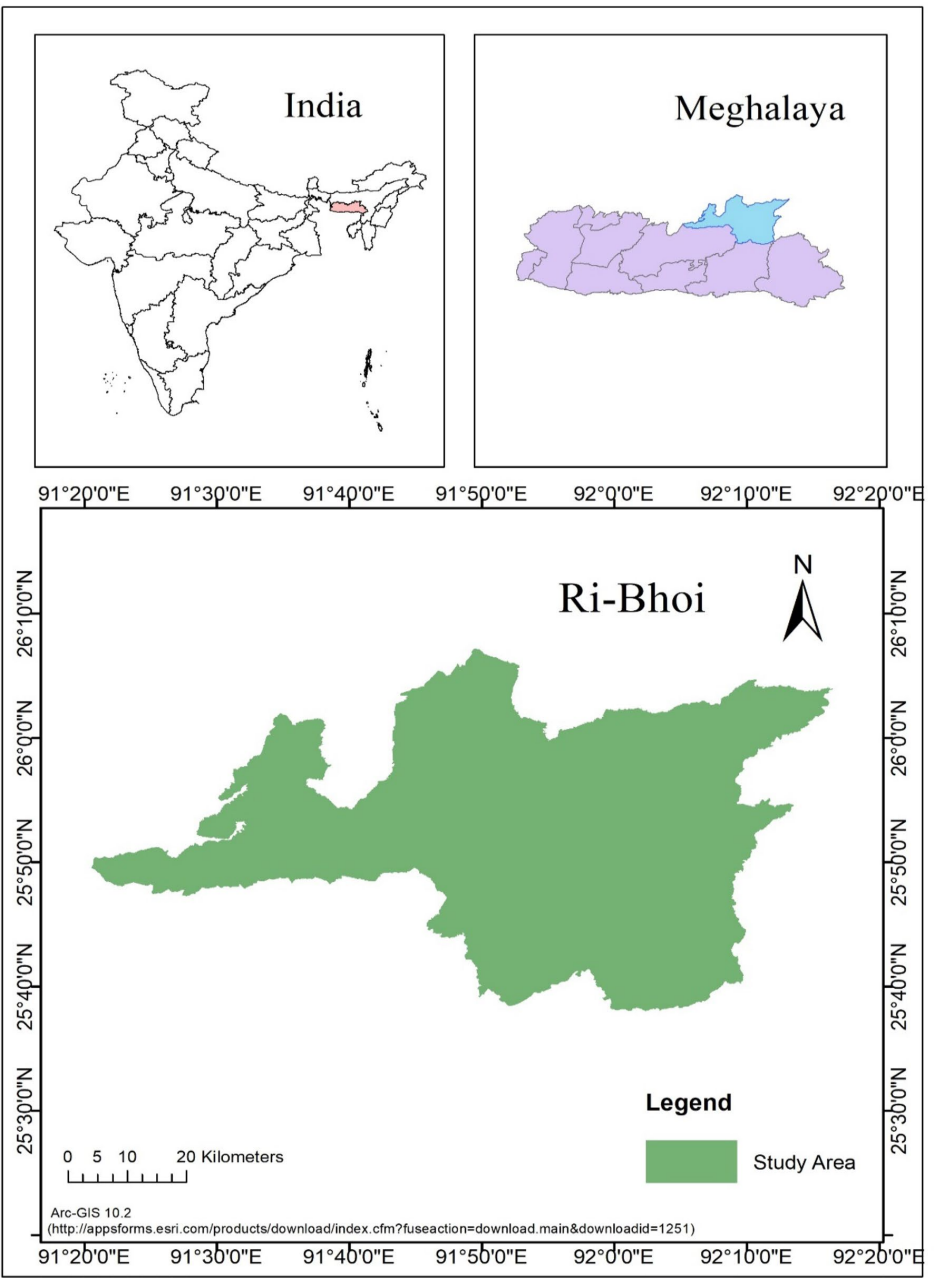
Study area Ri-Bhoi district is generated as per the selection of colour notation on base map of India, Meghalaya, and Ri-bhoi district in Arc-GIS 10.2. (https://appsforms.esri.com/products/download/index.cfm?fuseaction=download.main&downloadid=1251).

Ri-Bhoi district of Meghalaya lies between 90°55″15 to 91°16″ latitudes and 25°4″ to 25°2″ longitudes. It is bounded on the north by Kamrup district and on the East by Jaintia Hills and Karbi Anglong district of Assam and on the West by West Khasi Hills district (Fig. 1). In the south eastern part low hills are found to occur with valleys. The rivers in this area include Umtrew, Umsiang, Umran and Umiam. There is a marked relationship between elevation and slope in this area. Many patches in the valleys of main streams in the low elevated areas of northern borderlands have sloppy land below 15° of slope. On the other hand, in the higher elevated areas of Umiam valley, slope gradient ranges between 30° to 45° in most areas. However, the moderate elevated areas of central part have moderate slopes between 15° and 30°.

### Land use and land cover

Land use and land cover has also major influences on soil erosion^25^. Hence, it was planned to determine the erodibility indices on land uses basis. The district map was delineated from LISS IV image and land use land cover of the district has been determined as per the following procedure:
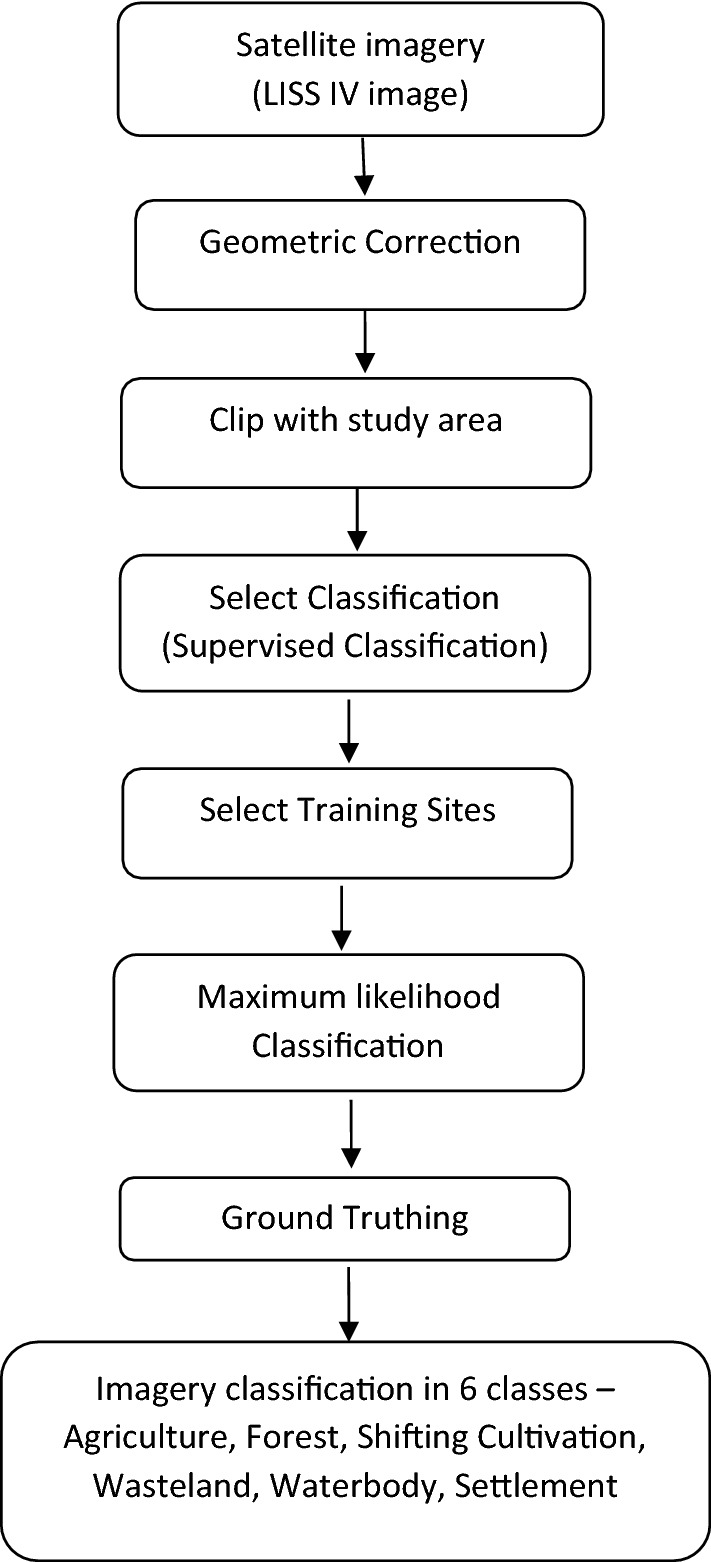


### Sample points

**Figure 2:**
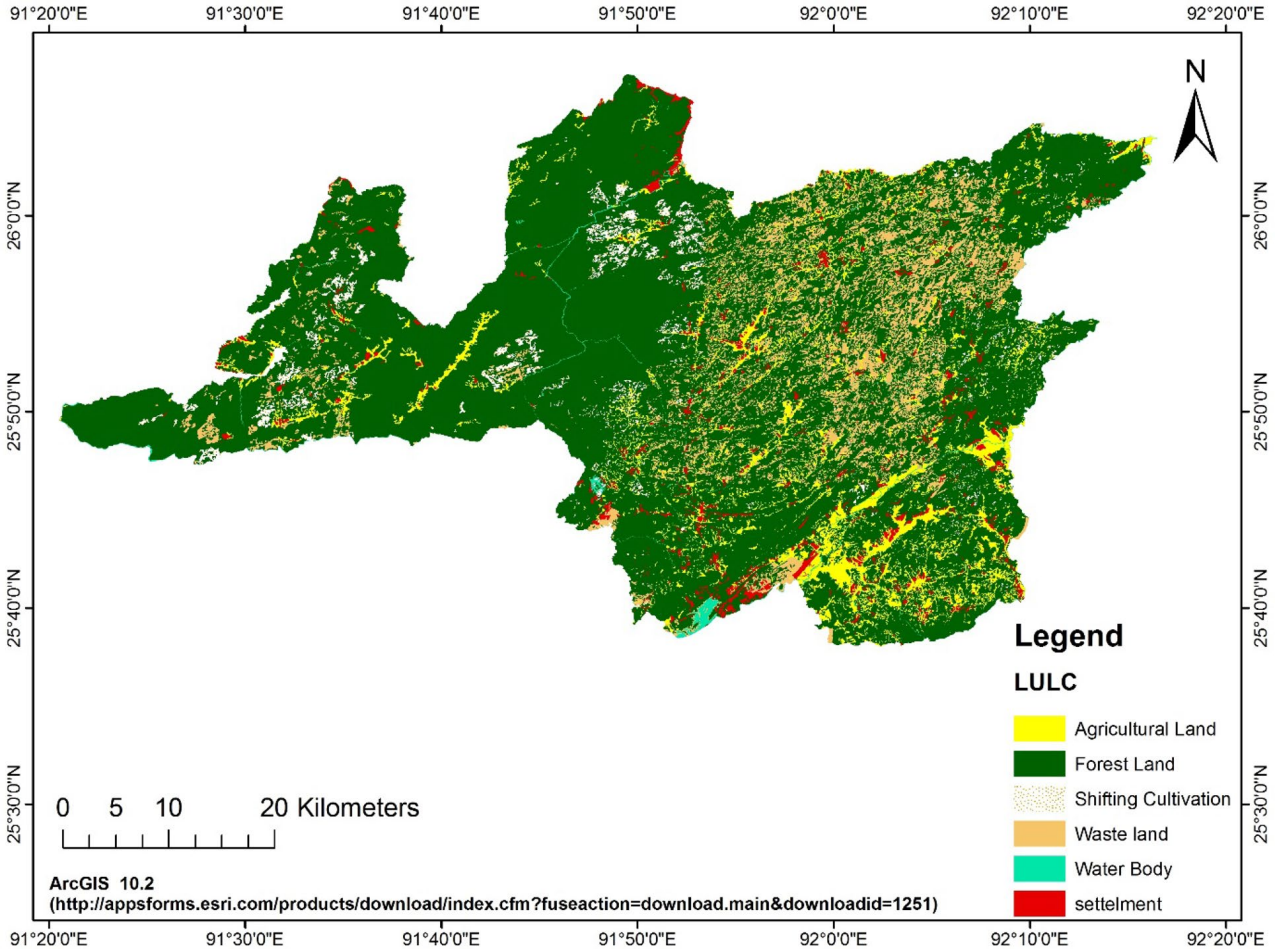
LULC map of the study area. (https://appsforms.esri.com/products/download/index.cfm?fuseaction=download.main&downloadid=1251).

**Figure 3:**
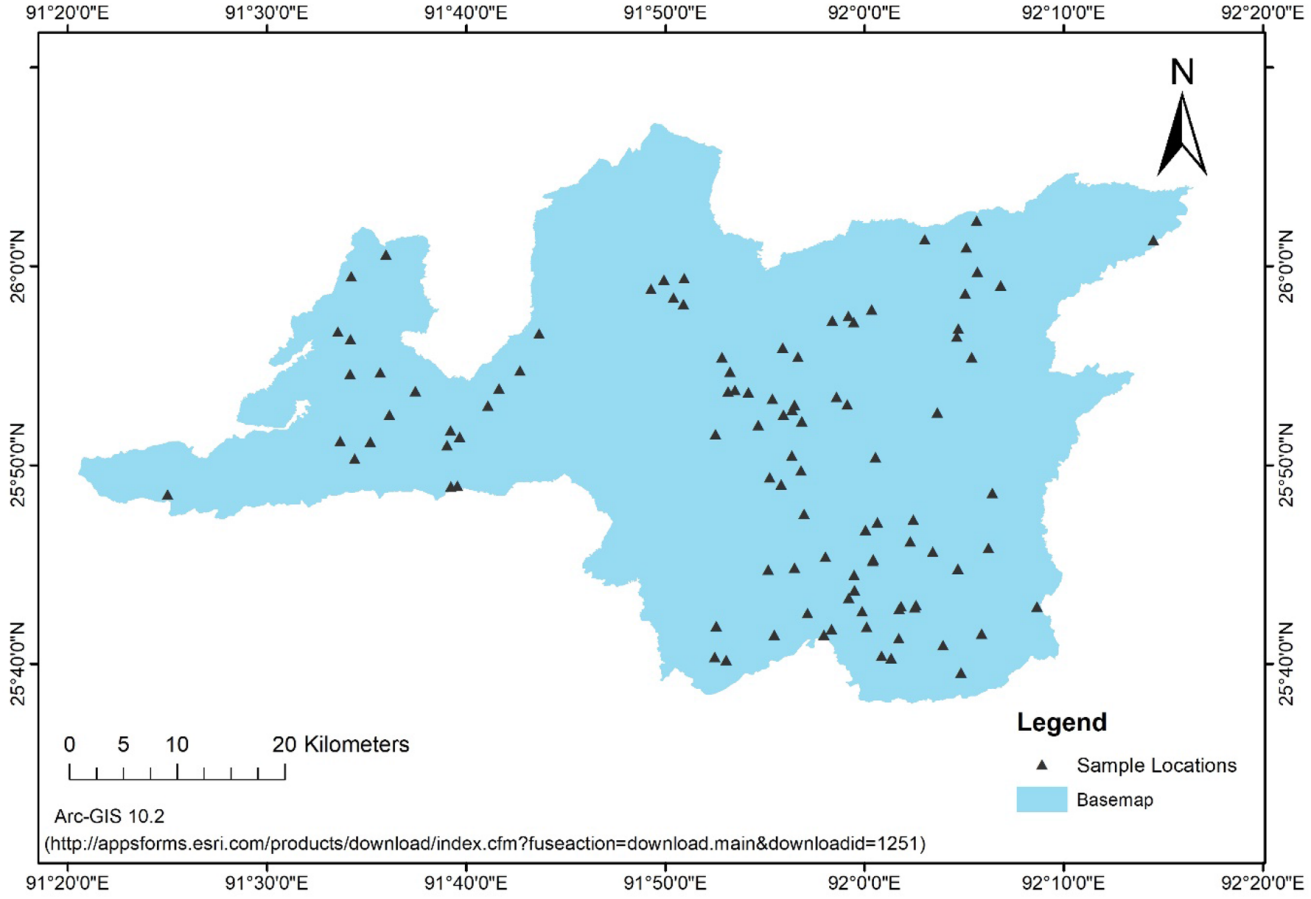
Sample points in Ri-bhoi district. Points of collected soil samples selected on the base map of Ri-bhoi with the help of geo-coded data of GPS. (https://appsforms.esri.com/products/download/index.cfm?fuseaction=download.main&downloadid=1251).

The Ri-bhoi district has four dominant types of land uses. They are agriculture, forest, *jhum* land (slash and burning agriculture field) and waste land (Fig. 2). Based on the land uses, 25 (twenty five) sample points in each of the land uses were randomly selected and geo-coded them with the help of hand held GPS. The sample points are shown for the whole district in Fig. 3. Composite soil samples to the depth of 15 cm (0–15 cm) around the selected sample points were collected for further analyses. Soil samples were analysed for texture with International Pipette method^26^ and organic carbon were determined by Walkely and Black method, permeability and structure classes were determined from the soil survey manual^8^.

### Clay ratio (CR) and modified clay ratio (MCR)

The clay ratio, is the estimation of amount of binding agent clay which tightly bind the soil particles, hard to detach the particle by the external forces in the presence of higher amount of clay particles^27^. Minimum amount requirement of clay for any kind of interpretation is 10%^22^. CR is inversely related to soil erodibility. Earlier studies stated the correlations among soil properties which reveals that modified clay ratio is also another index for erodibility^28, 29^. The clay ratio and modified clay ratio were obtained by the Eqs. (2) and (3) as shown below:

2 $${\text{CR}} = \left( {\% {\text{sand}} + \% {\text{silt}}} \right){/}\% {\text{clay}}$$

3 $${\text{MCR}} = \left( {\% {\text{sand}} + \% {\text{silt}}} \right){/}\left( {\% {\text{clay}} + \% {\text{OM}}} \right)$$

where values of percent sand, silt, clay and organic matter in the soil samples.

### Critical level of soil organic matter (CLOM)

Critical level of organic matter (CLOM) is also an indicator of erosion susceptibility. It is related to soil aggregate formation capability which offers resistance to soil erosion. If the value of CLOM is less than 5%, it is indicated that soil loses its structure and becomes highly susceptible to erosion. Soil is said to be moderately susceptible to erosion if the value lies between 5 and 7%. CLOM value more than 9% indicates that soil is stable and offer more resistance to erosion^23^.

CLOM is calculated with the Eq. (4).

4 $${\text{CLOM}} = \frac{OM}{{Clay + Silt}}$$

### Soil erodibility (K) index

The soil erodibility index or the K factor is defined as the rate of soil loss per unit of R (rainfall erosivity index based on EI_30_—30 min rainfall intensity energy causing erosion) on a unit plot and indicates the relative ease at which the soil is detached and transported^8^. Soil erodibility is mainly a function of texture, organic matter (OM) content, structure and permeability and can be determined based on the Eq. (5)^8, 30^.

5 $${1}00\;{\text{K}} = \frac{{2.1 M^{1.14} \left( {10^{ - 4} } \right)\left( {12 - OM} \right) + 3.25\left( {s - 2} \right) + 2.5 \left( {p - 3} \right)}}{7.59}$$

where K = erodibility factor in t ha h (ha MJ mm)^−1^; M = particle size parameter = (% silt + % fine sand) *(100 − % clay); OM = organic matter (%); s = soil structure class; p = permeability class.

The division by 7.59 gives values in SI units of t ha (ha MJ mm)^−1^.

### Interpolation of sampled data

Soils were sampled from four dominant land uses and 25 points were randomly selected in each of the land uses. The sample points were geo-coded and presented in the Fig. 3. The point information needs to be analysed so that the properties can be presented in map forms. Spatial data in GIS (Arc-GIS 10.2) platform are commonly interpolated in two methods viz*.* Kriging and Inverse Distance Weighting (IDW). Kriging is considered to be the best linear unbiased predictor and its prediction error variances are minimised^31^. Most of the soil physical properties, unlike truly stochastic parameters, do not vary randomly. The spatial variation of the properties is found to be gradual and are auto-correlated. Kriging takes care of both variance and co-variance of the sampled data. Therefore, the authors decided to adopt ordinary kriging for interpolation of the point data to transform them into raster forms.

#### Kriging

Daniel Krige developed empirically a statistical method to predict ore grade from a spatially correlated data in gold mines of South Africa in early fifties which has been formalized in sixties by Matheron^31^. Now Kriging has been applied in all discipline where spatially correlated samples are to be analysed. This has been considered as the Best Linear Unbiased Predictor (BLUP). In geo-statistics, often it is necessary to predict values in the intervening places and Kriging gives it in the most unbiased way with minimum variance^31^. Kriging techniques can be used to describe model spatial patterns, predict values at unmeasured locations, and assess the uncertainty associated with a predicted value at the unmeasured locations^31^. Ordinary kriging in original formulation by Matheron^31^ is the most popular, and it serves well in most situations. It is often regards as the ‘work-horse’ in geo-statistics. Kriging solves a set of linear equations, known as the kriging system, which contain semi variances drawn from a fitted variogram function.

The equations for ordinary kriging are given below^31^:

If z(x_i_), i = 1,2,3,…N, are observed values of variable z at points x_1,_ x_2_, … x_N_, where in two dimension x_i_ ≡ {x_i,1_, x_i,2_}^T^. Any new point x_0_ can be predicted Z by

6 $$Z\left( {x_{0} } \right) = \mathop \sum \limits_{i = 1}^{N} \lambda_{i} z\left( {x_{i} } \right)$$

Λ_i,1_ = 1,2,…N, are the weights chosen to minimise the prediction error variance by solution of the following set of equations:

7 $$\begin{aligned} \mathop \sum \limits_{i = 1}^{N} \lambda_{i} \gamma \left( {x_{i} - x_{j} } \right) + \psi \left( {x_{0} } \right) & = \gamma \left( {x_{j} - x_{0} } \right)\;for\; all \;j \\ & \mathop \sum \limits_{i = 1}^{N} \lambda_{i} = 1 \\ \end{aligned}$$

Here γ (x_i_ − x_j_) is the semi-variance between data points i and j. γ (x_j_ − x_0_) is the semi-variance between data point j and the target point x_0_ and $\psi \left( {x_{0} } \right)$ is a Lagrange multiplier introduced for the minimization of the error variance.

Kriging is used to generate CR, MCR and K-factor map by the following procedure.
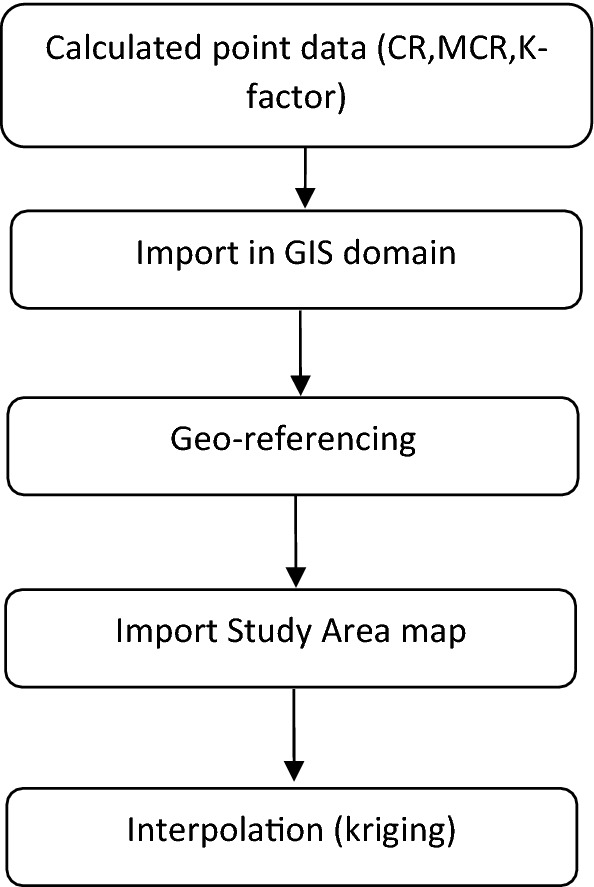


## Results and discussion

### Land use and land cover of the district

Ri-bhoi district of Meghalaya has multiple land uses including many sub-categories of agriculture and forest land covers. In the present study only four major land uses were considered (Fig. 2). Out of 2,359.67 km^2^ of total geographical area, forest constituted with 77.15%, agricultural land constituted with 8.11%, *jhum* area had 2.28% and wasteland was of 9.35%. Ri-bhoi, being a hilly district, most of the area fell under forest. Rice is the main crop both in agricultural and *jhum* areas. Majority of the agricultural area belonged to the practice of mono-crop, the valleys near the major stream is covered under vegetables. Baring forest covered area, the soil is expected to be manipulated with anthropogenic activities and falls under the major concern of soil erosion. Settlement constituted with 2.39% and water bodies made 0.70% of area. These two land uses were not considered for the study.

### Particle size distribution

The erodibility is low for clay-rich soils with a low shrink-swell capacity because clay particles mass together as soil aggregate by the cohesiveness and they are difficult to detach^32^ but once the clay particles are detached they are easily transported in suspension until they reach the stagnant water. The present study revealed that the clay had negative correlation with soil erodibility in the all land use types *i.e.* agriculture, *jhum*, forest, and wasteland. The higher avg. clay content value was observed in wasteland 37.36% followed by *jhum* (34.14%) and agriculture (32.95%), with minimum in the forest land 30.76% (Table 1). The clay content varies in LUS because of the parent material, mineral characteristics and weathering processes. It was reported that there’s a negative significant correlation between the amounts of clay and soil erodibility factor^33^. Clay content is used for determining soil erodibility^18^ and defined that soil with 9–30% of clay is taken as the most erodible. The least resistant particles in size separates are silts and fine sands^11^. Soils with 40–60% silt are highly susceptible to erosion^18^. Silt was significantly correlated with the erodibility in all LUS of study area. Silt was higher in the wasteland with an avg. value of 32.66% (17–53%), it was 32.17% (15.5–46.65%), 28.86% (14.5–38.5%) and 26.64% (10.5–45.5%) in agriculture, forest and *jhum* land uses, respectively. It was observed that six locations within wasteland, two locations within agriculture and three locations within *jhum* land uses had silt content more than 40% indicating susceptibility of erosion. No location in forest LU was considered to be susceptible to erosion with respect to silt content. Fine sand was also showing the positively significant correlation with soil erodibility. Fine sand content in agriculture, *jhum,* forest and wasteland land uses were 13.93%, 13.43%, 13.01% and 11.58%, respectively. Large particles such as sand are erosion resistant because of the force required to displace them is more compare to silt and fine sand. It was observed from the results that the sand was negatively correlated with the erodibility in agriculture, *jhum *(Table 2). The sand content were found to be 32.17%, 18.39%, 27.37% and 37.96% in agriculture, *jhum*, forest and wasteland, respectively.

**Table 1:** Soil properties of different land uses.

Land use	OM (%)	Silt (%)	Fine sand (%)	Clay (%)	K-factor
Agriculture	3.11 ± 0.08b	32.17 ± 1.88b	13.93 ± 1.14a	32.95 ± 2.29ab	0.25 ± 0.02a
*Jhum*	3.20 ± 0.09b	26.64 ± 1.61a	12.43 ± 0.89a	34.14 ± 1.61ab	0.20 ± 0.01a
Forest	3.19 ± 0.08b	28.86 ± 1.92ab	13.01 ± 0.82a	30.76 ± 2.18a	0.22 ± 0.02a
Waste land	2.67 ± 0.08a	32.66 ± 2.02b	11.58 ± 0.80a	37.36 ± 2.31b	0.23 ± 0.02a

Different letters used for the indication of differences among soil properties of major Land uses.

**Table 2:** Correlation of K-index with determinant factors in the district.

	K-index	Sand	Silt	Clay	Om	Fine sand	Soil permeability
K-index	1						
Sand	− 0.300**	1					
Silt	0.798**	− 0.639**	1				
Clay	− 0.612**	− 0.545_**_	− 0.217^27^	1			
Om	− 0.340**	0.126	− 0.282**	0.109	1		
Fine sand	0.648**	− 0.296**	0.288**	− 0.318**	− 0.037	1	
Soil permeability	− 0.259**	0.648**	0.059	− 0.768**	0.069	− 0.046	1

Correlation coefficient (r) = 0.200, 0.0250 at *P_0.05_ **P_0.01_, n − 2 = 98.

### Organic carbon

Soil organic carbon was observed to be higher in *jhum* LUS with average of 3.20% followed by forest LUS with 3.19%, agriculture 3.11% and lowest observed in wasteland 2.67%. Higher organic carbon was reported in forest LUS^34^ followed by agriculture and minimum in wasteland. Similarly organic carbon^35^ was reported higher in *jhum* and forest land. Silt, clay, residue turnover from biomass and its decomposition rate are the critical factors in SOC build up in soil^36^. It might be attributed to the deposition of plant litter falls and death decay of above ground biomass of natural vegetation. The slash and burring procedure of the *jhumming* might help in the exposure of OC in the terrestrial ecosystem. Variation in organic carbon in soils under various land uses might be due to varying leaf litter and their rate of decomposition^30^. Similar result saying that SOC of Natural Forest soils are found to be higher than in the other land use types. Maintaining SOC levels in tropical soils was found to be more difficult because of its rapid oxidation under prevailing high temperatures.

### Clay ratio (CR)

**Figure 4:**
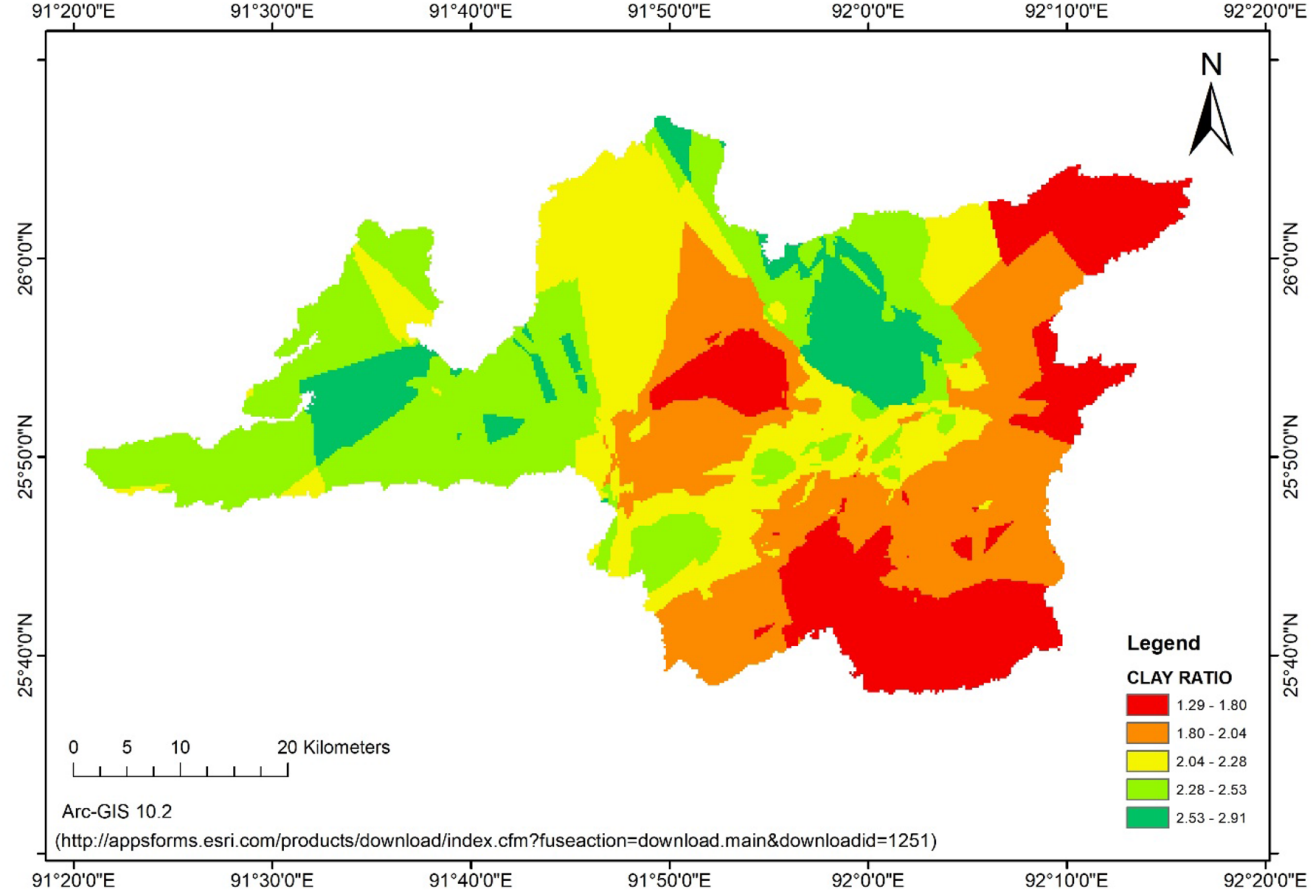
Clay Ratio (CR) of Ri-bhoi district. (https://appsforms.esri.com/products/download/index.cfm?fuseaction=download.main&downloadid=1251).

Clay ratio (CR) in agriculture, *jhum*, forest and waste land ranged from 0.63–6.40, 1.0–4.26, 1.02–8.52 and 0.72–5.25, respectively. The average values of CR in the land uses were 2.74, 2.39, 2.10 and 1.97, respectively for forest, agriculture, *jhum* and wasteland. The higher value of CR for forest soil was due to higher content of sand (40.38%). The lower CR value in wasteland was due to the lower sand content in the soils of wasteland compared to other land use systems. CR for overall Ri-Bhoi district ranged from 0.63 to 8.52 with an average value of 2.30. The Fig. 4 represents the Clay Ratio map of Ri-bhoi district. Though higher CR values indicate susceptibility to erosion, since this is a ratio between the erosion susceptible primary particles to clay, the CR values of different land uses could not give any conclusive understanding of its relationship to the erosion proneness. It was stated that for any meaningful interpretation the clay content should be more than 10%^22^. All the samples in the study had clay content more than 10%, but due to absence of any scale for erosion proneness, conclusive interpretation could not be drawn.

### Modified clay ratio

**Figure 5:**
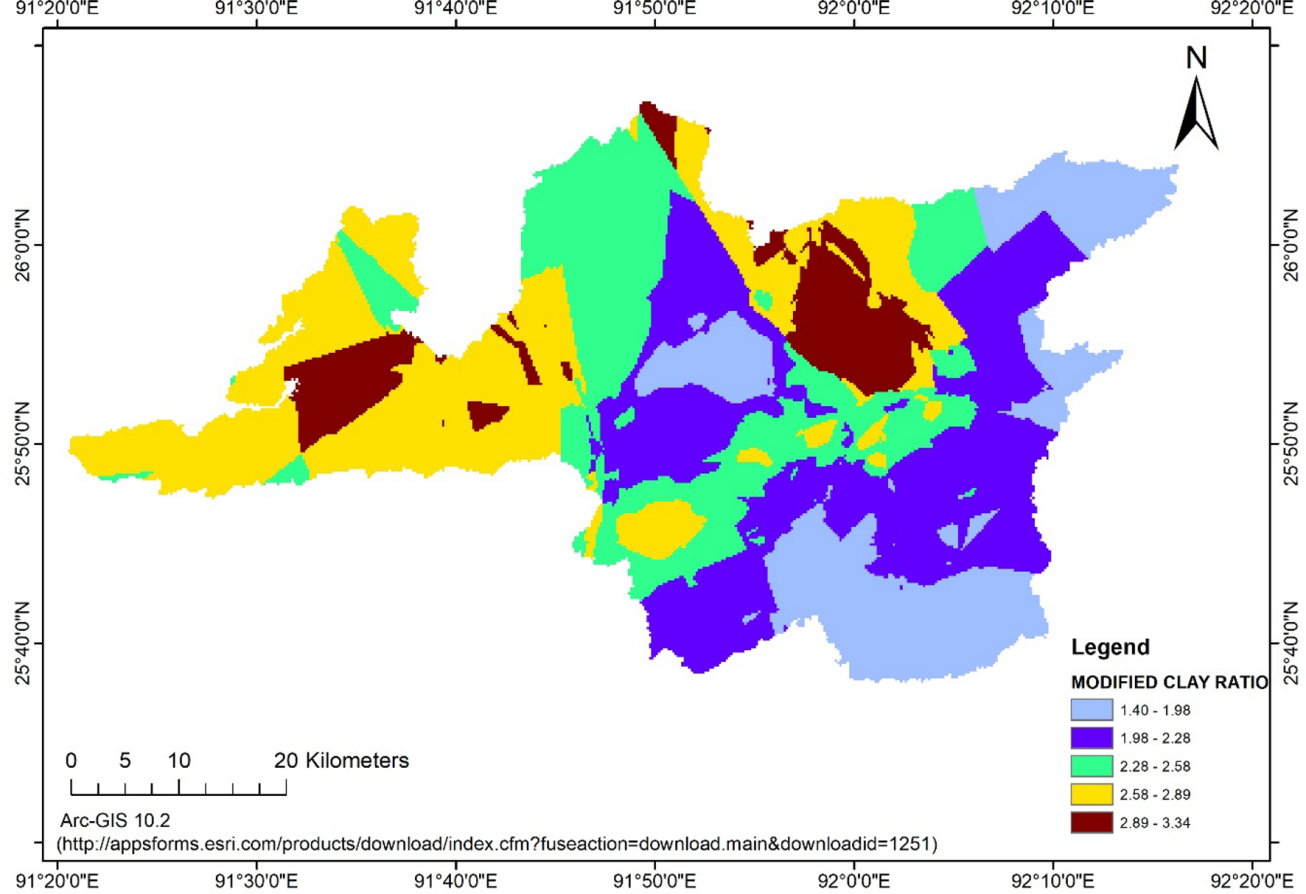
Modified Clay Ratio (MCR) of Ri-bhoi district. (https://appsforms.esri.com/products/download/index.cfm?fuseaction=download.main&downloadid=1251).

Modified clay ratio for agriculture, *jhum*, forest and wasteland were observed in the range of 0.61–5.17, 0.94–3.57, 0.96–6.84 and 0.68–4.53, respectively. The average value of modified clay ratio was observed higher in forest land use (2.41). This is slightly less than the clay ratio because organic matter was added in the denominator. The trend of MCR was observed in the similar manner of CR and in the decreasing order for the land uses of agriculture, *jhum* and wasteland as 2.13, 1.89, 1.81, respectively. The MCR for Ri-Bhoi district of Meghalaya was observed in the range of 0.61–6.84 with the average value of 2.06. The MCR values of Ri-bhoi district is presented in Fig. 5. Similar to the CR, MCR also failed to give conclusive interpretation about the erosion proneness of the soil according to the land uses. However, as the average values of MCR were not so high, it could be inferred that soils in the district was not highly susceptible to erosion.

### Critical level of soil organic matter (CLOM)

The results indicated that the values of CLOM for the district ranged from 4.72 to 16.56. Out of 100 samples, only one sample had CLOM value less than 5 and rest 99 samples had values more than 5 indicating that the soils of the district were moderate to stable soil structure and offer resistance to erosion. Average CLOM values were 8.84, 9.46, 9.41and 6.71 for agriculture, forest, *jhum* and wasteland, respectively. Land use wise CLOM values ranged from 5.70 to 16.56 for agriculture, 574 to 15.01 for forest, 6.31 to 12.52 for *jhum* and 4.72 to 9.69 for wasteland. Soils were moderately susceptible to erosion in 60%, 40%, 48% and 88% for agriculture, forest, *jhum* and wasteland land uses. Further, 40%, 60%, 52% and 8% locations in agriculture, forest, *jhum* and wasteland, respectively had very stable soil aggregates indicating the wasteland was relatively susceptible to erosion.

Soil aggregate stability is related to Soil Organic Matter. In the district, soil organic carbon ranged from 2.67 in wasteland to 3.20 in the forest land use which corroborate with the CLOM values indicating that soil stability positively correlated with the soil organic matter.

### Soil structure

The Eq. (5) requires soil structure classes. It considers 4 classes of soil structure and assigned codes 1–4 for the equation. Codes are viz*,* 1: very fine granular (1–2 mm mean aggregate diameter), 2: fine granular (2–3 mm), 3: medium or coarse granular (3–5 mm) and 4: blocky, plat or massive (> 5 mm)^8^ . Soils in agricultural land found to have 1 and 2 structural codes, *Jhum* had 1, 2 and 3, forest had 2, 3 and 4 and the wasteland had the same structural codes of *jhum* land. Agricultural land with repeated tillage for cultivation had finer soil aggregates and prone to easier detachment and displacement. Similarly, forest soils with very less disturbance had better soil aggregation and resistance to erosion. As wasteland had higher clay content (37.36%), had finer to coarser soil aggregates even though the soils were exposed to various degradation forces. The Eq. (5) does not use absolute values of Mean Weight Diameter and hence it was difficult to enumerate the exact influence on soil erodibility in relation to land uses.

### Soil permeability

There are six permeability classes used in Eq. (5). They are viz*.* 1: rapid (> 150 mm/h), 2: moderate to rapid (50–150 mm/h), 3: moderate (12–50 mm/h), 4: slow to moderate (5–15 mm/h), 5: slow (1–5 mm/h) and 6: very slow (< 1 mm/h).

Permeability and stability of the soil structure are two very important soil properties, which affect its erodibility factor^15^. Agriculture lands have the permeability classes from 2,3,4,5 and 6. In *jhum* the permeability classes vary from 3, 4, 5 and 6. The permeability class for forest land was observed in the same trend as agriculture areas and similar trend for wasteland was observed as in case of *jhum* areas. Soil permeability class-1 was absent in all major LUS. Permeability class-1 has higher rate of infiltration with very low runoff potential and lowest rate of infiltration was derived for the class-6^8, 37^. Lower permeability class values allows the water to enter in the soil rapidly which reduces the runoff and higher values of permeability classes shows the slow rate of infiltration which may increase the rate of runoff in the soil which allows the light texture particles viz*.,* silt and fine sand to get affected with the erosive forces. Agriculture and forest areas had permeability class ranging from 2 to 6 and hence expected to have spatially variable infiltration. In the areas with 5 and 6 permeability classes are expected to have very high runoff potential and hence soil erosion. As compared to agriculture and forest, *Jhun* and wasteland are more susceptible to soil erosion due to higher overland flow with higher permeability classes.

### Soil properties of different land uses and their impact on erodibility

The soil physical properties, that influence the soil erodibility index, were compared according to the land uses with the premise that land uses are many a times decided by the soil physico-chemical properties and the soil texture was expected to be varying according to the land uses. The average organic matter in the soils was varying from 2.67% in wasteland to 3.20% in *jhum* land. The organic matter content in agriculture and forest land was not significantly different from that in *jhum* land but statistically different from wasteland (Table 1). The silt content was found to be similar in forest and *jhum* land and the silt content in forestland was at par with agriculture and wasteland. The content of fine sand was statistically similar for all the land uses which was ranging from 11.58% to 13.93%. Clay content was found to be different according to the land use types. Wasteland was having highest clay content with average value of 37.36%, which was statistically different from forest land but at par with agriculture and *jhum* land.

The information of soil physical properties and the indices such as CR, MCR, CLOM, etc. gave only inference about the soils proneness to erosion. This is a qualitative information and not suitable for any application for soil erosion assessment models. However, this qualitative information may be useful in case of prioritization of soil conservation measures^24^ and hence their importance cannot be undermined. However, K-index as given in USLE^8^, gives quantitative values of soil erodibility and can be used directly for assessment of soil loss.

### K-index

Soil erodibility values in agriculture LU were ranging from 0.08 to 0.42 with an average value of 0.25 ± 0.02 which was the highest in the district. In *jhum* LUS these values ranged from 0.08 to 0.42 with an average of 0.20 ± 0.01 which was lowest among all major land uses. For forest LUS, values were ranging from 0.09 to 0.40 with an avg. of 0.22 ± 0.02. The erodibility index values in wasteland were observed in the range of 0.10–0.34 with an avg. value of 0.23 ± 0.02.

### Soil erodibility maps

**Figure 6:**
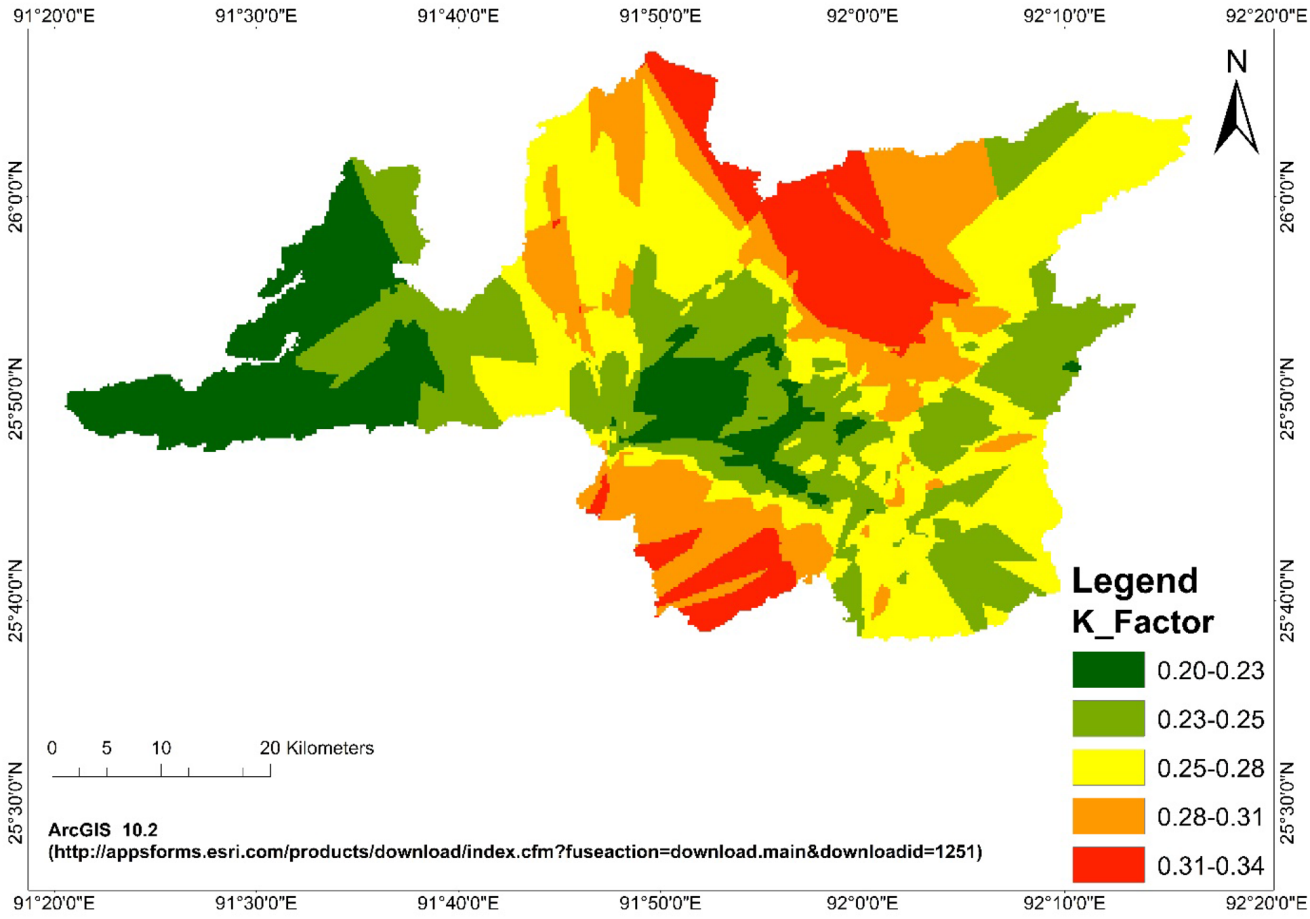
Soil Erodibility (K) Factor map of Ri-bhoi district. (https://appsforms.esri.com/products/download/index.cfm?fuseaction=download.main&downloadid=1251).

The values of soil erodibility of the geo-coded sampling points were interpolated in GIS environment through kriging^31^ and soil erodibility maps according to land use systems were prepared and presented in Fig. 6. The higher K value in agriculture was due to the higher amount of silt and fine sand content. Silt and fine sand lacks adhesion properties and if moisturized, becomes easily broken and transported, having an increased impact on soil erodibility^38^. Lowest erodibility observed in the *jhum* LUS due to the higher organic matter that allowed soil fractions to make greater aggregate stability in *jhum* LUS and sand fraction had also played role for reducing the erodibility in *jhum* LUS because of greater force requirement to displacement the particles. In case of forest LUS, clay content and sand content had played dominant role to reduce the erodibility. Soil erodibility was negatively correlated with sand in agriculture, *jhum* and forest LUS but no trend was observed in wasteland because of clay fraction was dominant than other soil fractions. Sand had also shown the negative correlation with the erodibility, increase of sand content reduced the erodibility. In case of wasteland higher correlation was observed with clay. Similar trend was observed in earlier works^4^. Permeability had also shown negative correlation with erodibility which means in all LUSs which reduced the rate of runoff.

The K-index map was prepared for the district with five ranges of index values viz. 0.20–0.23, 0.23–0.25, 0.25–0.28, 0.28–0.31 and 0.31–0.34. Out of total area of 2,428.89 km^17^ of the district most of the area 72.52% had soils with K-index less than 0.28. It is seen from the Fig. 6, that forest and *Jhum* area mainly fall under these three categories. K-index ranging from 0.28 to 0.34, are mainly spread over agriculture and wasteland areas spreading towards northern and southern parts of the district and constitute 27.48% area of the district. The K-index map with these five categories of K-index values has been prepared with very small interval of 0.03 and represented with distinct colour coded polygons. This will enable to pick up K-index values easily from the polygon areas with the average values of the range interval.

### Erodibility indices (K-index) vis-a-vis soil physical properties and land uses

Soil texture is an inherent property and does not change with land uses. However, erodibility indices are dependent on not only soil textures but also on the presence of OM, soil permeability and soil structures. It was observed from the Table 1 that K-index varied from 0.20 ± 0.1 (*Jhum*) to 0.25 ± 0.2 (agriculture) though difference among the K-indices corresponding to the land uses were not statistically significant. Average compositions of the primary particles of the soil (sand, fine sand, silt and clay) did not have any correlation with the land uses. Agriculture LU had higher silt and fine sand in comparison to the soils of other land uses as a result the soils in agricultural areas had higher K-index values. The presence of higher clay could not offset the effect of silt and fine sand. The presence of lower organic matter in agricultural LU in comparison to forest and *Jhum* LU also attributed to higher K-index. Wasteland had lowest amount of OM (2.67 ± 0.08) and highest amount of silt per cent (32.66 ± 2.02). Even though the clay per cent (37.36 ± 2.31) was the highest in wasteland soil among the four land uses, the K-index values on wasteland were relatively higher with an average value of 0.23 ± 0.02. From Table 1, it was evident that the soils in *jhum* area were less erodible than the soils in agriculture and wasteland LU. This is however against the popular belief that *jhum* soils are susceptible to soil erosion. This may be attributed to the content of higher organic matter (3.20 ± 0.09) in *jhum* soils.

The pairwise correlation of K-index with the soil texture and organic matter revealed (Table 2) very strong positive correlation of K-index with silt (0.798) and fine sand (0.648) and significant negative correlation with clay (− 0.612) and organic matter (− 0.340). K-index was also negatively correlated with soil permeability (− 0.259). These correlations corroborated the general understanding of the soil erodibility vis-a-vis soil texture, organic matter and permeability. Soil structural codes were not analysed for correlations as it was a qualitative range used in the Eq. (5).

### Spatial variability of K-index

The greater variability of soil erodibility (K-index) was in *jhum* land use (0.08 to 0.42) and forest land use (0.09 to 0.4) as compared to that (0.1 to 0.34) of agricultural land use and wasteland. These findings clearly indicated that spatial variability of soil texture, organic matter; soil permeability and soil structure were more in *jhum* and forest land use than in agricultural and wasteland. The content of silt was found to be the strongest determinant of K-factor for agricultural, *jhum* and forest land uses, whereas the content of clay was for wasteland. Soil structure seemed to play more significant effect on K-factor of forest LU than that of agricultural, *jhum* and waste land uses.

## Conclusion

The indices like Clay Ratio, Modified Clay Ratio, and Critical Level of Soil Organic Matter could reveal the information about the proneness of soil to erosion in qualitative manner and helped in prioritizing any area as susceptible, moderately stable or stable for soil erosion. Clay Ratio for the district was ranging from 0.63 to 8.52 with an average value of 2.30. CR was found to be lowest in case of wasteland. MCR for the district was also observed in the range of 0.61–6.84 with the average of 2.06. Both the indices had similar trend. With CR, MCR values, it was found that wasteland had higher resilience whereas it was reverse as per K-index. Excessive reliance on primary particles of soil without considering soil structures, permeability and organic matter may become misleading at times. In order to make a quantitative assessment of soil erosion, the K-index was found to be better which incorporate all the soil physical properties which are the main determinants of soil erodibility. K-index revealed that agriculture followed by wasteland were prone to erosion whereas *jhum* followed by forestry were less affected by erosion due to prevailing soil texture and higher organic matter. The soil erodibility (K-index) index map generated in GIS platform through interpolation for different land uses will provide K-index values of any location of the district which can be directly used by field workers for estimation of soil erosion at any place with given given land use, conservation practice and rainfall erosive force.
